# Infection with *Porphyromonas gingivalis* Exacerbates Endothelial Injury in Obese Mice

**DOI:** 10.1371/journal.pone.0110519

**Published:** 2014-10-21

**Authors:** Min Ao, Mutsumi Miyauchi, Toshihiro Inubushi, Masae Kitagawa, Hisako Furusho, Toshinori Ando, Nurina Febriyanti Ayuningtyas, Atsuhiro Nagasaki, Kazuyuki Ishihara, Hidetoshi Tahara, Katsuyuki Kozai, Takashi Takata

**Affiliations:** 1 Department of Oral and Maxillofacial Pathobiology, Institute of Biomedical and Health Sciences, Hiroshima University, Hiroshima, Japan; 2 Department of Pediatric Dentistry, Institute of Biomedical and Health Sciences, Hiroshima University, Hiroshima, Japan; 3 Center of Oral Clinical Examination, Hiroshima University Hospital, Hiroshima University, Hiroshima, Japan; 4 Department of Microbiology, Tokyo Dental College, Tokyo, Japan; 5 Department of Cellular and Molecular Biology, Institute of Biomedical and Health Sciences, Hiroshima University, Hiroshima, Japan; Boston University, United States of America

## Abstract

**Background:**

A number of studies have revealed a link between chronic periodontitis and cardiovascular disease in obese patients. However, there is little information about the influence of periodontitis-associated bacteria, *Porphyromonas gingivalis* (*Pg*), on pathogenesis of atherosclerosis in obesity.

**Methods:**

*In vivo* experiment: *C57BL/6J* mice were fed with a high-fat diet (HFD) or normal chow diet (CD), as a control. *Pg* was infected from the pulp chamber. At 6 weeks post-infection, histological and immunohistochemical analysis of aortal tissues was performed. *In vitro* experiment: hTERT-immortalized human umbilical vein endothelial cells (HuhT1) were used to assess the effect of *Pg/Pg*-LPS on free fatty acid (FFA) induced endothelial cells apoptosis and regulation of cytokine gene expression.

**Results:**

Weaker staining of CD31 and increased numbers of TUNEL positive cells in aortal tissue of HFD mice indicated endothelial injury. *Pg* infection exacerbated the endothelial injury. Immunohistochemically, *Pg* was detected deep in the smooth muscle of the aorta, and the number of *Pg* cells in the aortal wall was higher in HFD mice than in CD mice. Moreover, *in vitro*, FFA treatment induced apoptosis in HuhT1 cells and exposure to *Pg*-LPS increased this effect. In addition, *Pg* and *Pg*-LPS both attenuated cytokine production in HuhT1 cells stimulated by palmitate.

**Conclusions:**

Dental infection of *Pg* may contribute to pathogenesis of atherosclerosis by accelerating FFA-induced endothelial injury.

## Introduction

Obesity is an independent risk factor for the development of atherosclerosis. Atherosclerosis, characterized by the accumulation of lipids and fibrous elements in the aortal tissues, is the most important contributor to the progress of cardiovascular disease (CVD) [1]. CVD, formerly the leading cause of death and illness in developed countries, has also become a more serious health problem worldwide [2]. While atherosclerosis was formerly considered a simple and relatively passive lipid storage disease, the past decades have seen an increased interest in the role that chronic inflammation may play in triggering atherosclerosis [3]. According to the “response to injury” hypothesis by Ross (1999), the early stage of atherosclerosis is initiated by a chronic inflammatory response of the arterial wall to endothelial injury, which is called endothelial dysfunction, and is caused by hyperlipidemia, elevated plasma homocysteine concentrations, hypertension, and infectious microorganisms [4]. This dysfunction consequently leads to imbalanced blood vessel regulation and enhanced leukocyte adhesion through up-regulation of adhesion molecules, the synthesis of proinflammatory and prothrombotic factors, and oxidative stress [5].

Recently, epidemiological and interventional studies have revealed a link between atherosclerosis and bacterial infections, including *Chlamydia pneumoniae*, *Helicobacter pylori*, and *Porphyromonas gingivalis* (*Pg*) [6]–[10]. Periodontitis is a chronic infectious/inflammatory disease caused by periodontal pathogens, and *Pg* is one of the primary species related to both chronic marginal periodontitis and periapical periodontitis [11]–[13]. Several clinical trials have revealed an association between periodontitis and levels of systemic inflammatory markers and endothelial injury. However, to our knowledge, there is no report to date clarifying the relationship between *Pg* dental infection and endothelial injury in a diet-induced obese mouse model.

In the present study, we analyzed how dental infection by *Pg* affects endothelial injury, the early stage of atherosclerosis that occurs in obesity. To accomplish this, we employed an *in vivo* model where mice with *Pg-*induced periapical periodontitis were maintained on a normal chow diet (CD) or a high fat diet (HFD). Moreover, hTERT-immortalized human umbilical vein endothelial cells (HuhT1) were used to assess the effect of *Pg* whole cell, *Pg*-LPS and/or free fatty acid (FFA), *in vitro*. We report that HFD results in endothelial injury in mice aortal tissues, and *Pg* infection exacerbates this effect. In parallel, *in vitro* experiments similarly demonstrate that *Pg* whole cell and *Pg*-LPS up-regulates cytokine expression and increases apoptosis of FFA-treated HuhT1 cells. These findings suggest that dental infection by *Pg* promotes endothelial injury in the context of obesity.

## Materials and Methods

### Animal studies

This study was carried out in strict accordance with the recommendations in the Guide for the Care and Use of Laboratory Animals of the Hiroshima University Animal Research Committee and AVMA Guidelines on Euthanasia. The protocol described below was approved by the Committee on the Ethics of Animal Experiments of the Hiroshima University (Permit Number: A09-89). All mice were housed in a specific pathogen free facility in 12 hr light-dark cycles with access to water *ad libitum*.

A total number of 24 5-week-old male SPF *C57BL/6J* mice (Charles River Japan, Inc., Yokohama, Japan) were randomly divided into two groups, fed either a normal chow diet (CD) or a high fat diet (HFD-60; Oriental Yeast Co., Ltd., Tokyo, Japan). After establishment of the obese mouse model by 12 weeks of HFD feeding, mice were further divided into two subgroups (N = 6 each): Mice in the HFD-*Pg* group were infected by *Pg* (as detailed below), while the HFD-NC group served as the negative control without infection. Similarly, the CD group was subdivided and further treated to make CD-NC and CD-*Pg* groups.

### *Pg* infection

All surgeries were performed on mice under intraperitoneal anesthesia induced by pentobarbital sodium (1.62 mg/30 g; Kyoritsu Seiyaku Co., Tokyo, Japan) and atropine sulfate (12.5 ug/30 g; Mitsubishi Tanabe Pharma Co., Osaka, Japan). Surgeries were performed in biosafety cabinets of Hiroshima University Animal Facility, and maximum efforts were made to minimize postoperative pain and suffering. To establish dental infection of *Pg*, the pulp chambers of the maxillary first molars on the left and right sides were opened with a #1/2 round bur. After removing the coronal pulp, a small sterile cotton swab including 1 µl of absorbed bacterial suspension (10^7^ CFU of *Pg* W83 strain) was inserted into the pulp chamber, which was then sealed with Caviton (GC Co., Tokyo, Japan). Six weeks later, mice were euthanized and samples of maxilla and 5 mm length of aortal tissues from descending aorta at the level equivalent to heart were harvested. Tissues were stored at −80°C for further evaluation or processed as formalin-fixed paraffin embedded (FFPE) samples.

### Immunohistochemistry

Immunolocalization of *Pg* in the aortal wall was examined by using rabbit antiserum against *Pg* whole cell (1∶1,000 dilution). Anti-mouse CD-31 monoclonal antibody (1∶100 dilution; Dianova GmbH, Hamburg, Germany) was used for immunohistochemical detection of endothelial cells. Immunohistochemical staining was performed using a high polymer method (Histofine Max-PO: Nichirei Biosciences, Tokyo, Japan). Specificity was ascertained by substituting PBS or serum for each primary antibody. Sections were analyzed under light microscopy and photomicrographs obtained at 400x magnification.

### Terminal deoxynucleotidyl transferase dUTP nick end labeling (TUNEL)

To measure *in situ* apoptosis, TUNEL staining was performed on aortal tissues obtained from each group. Tissue samples were processed using an In Situ Apoptosis Detection Kit (Trevigen Inc., Gaithersburg, MD, USA) according to manufacturer’s instructions. Negative controls were obtained for each sample by omission of incubation with the TUNEL reaction mixture. Sections were analyzed under light microscopy and photomicrographs were obtained at 400x magnification.

### Histomorphometric analysis

Analysis was performed on 8 sections of aortal tissue per mouse. *Pg* colonies were immonohistochemically stained (as described above) and the number of brown pigments (i.e., *Pg* colonies) was counted under a microscope. The average number of colonies for each group was calculated. The ratio (%) of CD31-positive length per total length of the inner surface of the aortal wall was calculated after immunohistochemical staining (as described above). The total number of TUNEL-positive endothelial cells on was counted and normalized to the number of positive cells per 1 mm inner surface of aortal wall.

### Identification of *Pg* in aortal tissue by PCR analysis

Total DNA was extracted from mouse aortal tissue by DNeasy Tissue Kit (QIAGEN Science, Germantown, MD, USA) according to the manufacturer’s instructions. The *mgl* gene that encodes l-methionine-α-deamino-γ-mercaptomethane-lyase (METase) and specifically identifies *Pg* [14] was amplified by PCR. The primer pair used was 5′-GCTATCGAGAACGCCTTC-3′ (Forward) and 5′-GCAGTGCCATCTGCTTCT-3′ (reverse). Genomic DNA of the *Pg* W83 strain was used as a positive control template.

### Cell culture and treatment

hTERT-immortalized human umbilical vein endothelial cells (HuhT1) were used in this study. HuhT1 cells were previously established by transfection with human telomerase reverse transcriptase [15]. HuhT1 cells were maintained in HuMedia-EG2 growth medium with a commercial supplemental kit (Kurabo, Osaka, Japan). 1.5×10^5^ of HuhT1 cells were inoculated to a 35 mm collagen coated dish for the following experiments. 10 mM palmitate (Sigma, Hamburg, Germany) per 1% Bovine Serum Albumin (BSA) stock solution was prepared according to the method of Wobser [16]. FFA-free-BSA-treated cells were used as negative control. Before each treatment, medium was completely removed, cells were washed with PBS, and culture medium was replaced a fresh batch. Lipopolysaccharide of *Pg* (*Pg*-LPS, Strain ATCC 33277) was purchased from InvivoGen and a concentration of 100 ng/ml was used. Cells were maintained at 37°C in a normal atmosphere containing 5% CO_2_.

### Flow cytometry

Huht1 cells were plated on 60 mm dishes at a density of 4×10^6^. Cells were treated with 100 uM palmitate or 1 ug/ml Doxorubicin (Dox, positive control of apoptosis) for 12 h. PE Annexin V apoptosis Detection Kit1 (BD Pharmingen) was used according to the instruction of manufacture. In brief, cells were washed twice with ice-cold PBS, resuspended in 50 ul binding buffer, added Annexin V-PE (2.5 ul) and 7AAD (2.5 ul) and mixed gently. Incubation was performed at room temperature avoiding light for 15 min. Binding buffer (400 ul) was added to each sample and proceeded to flow cytometry analysis.

### Antibiotic protection assay

HuhT1 cells were pre-incubated with palmitate (50 µM) for 12 h. Before *Pg* infection, culture media were replaced by an antibiotic-free medium for at least 2 h, followed by gentle rinsing three times with PBS. Cells were exposed to *Pg* at a multiplicity of infection (MOI) of 100 for 2 h, and then incubated with metronidazole (200 µg/ml) and gentamicin (300 µg/ml) for 1 h to kill extracellular bacteria [17]. Cells were permeated with 1 ml of sterile, nonpyrogenic distilled water for 20 min and 100 µl of 10x diluent was plated on blood agar supplemented with 10% defibrinated sheep blood, hemin (5 µg/ml), and menadione (0.5 µg/ml), and cultured under anaerobic conditions (Anaeropack system, Mitsubishi Gas Chemical Co., Tokyo, Japan) at 37°C. The number of intracellular invaded *Pg* cells was determined by colony counting on blood agar plates.

### Cell counting

After 12 h pre-incubating with or without palmitate (50 µM), HuhT1 cells were treated with *Pg*-LPS (100 ng/ml) for an additional 12 h. The number of floating versus attached cells was determined by a cell counting machine (Z1, Coulter, Hialeah, FL, USA) in order to calculate the percentage of dead (floating) cells in the total number.

### RT-PCR

HuhT1 cells (1.5×10^5^ cells) were pre-incubated with palmitate (50 µM) for 12 h. After 1 h of treatment with *Pg*-LPS (100 ng/ml), total RNA was extracted from the cell pellets using the RNeasy Mini Kit (Qiagen, K.K., Tokyo, Japan) following the manufacturer’s instructions. RNA concentration and purity were determined by spectrophotometry. 1 µg of total RNA was used for cDNA synthesis using the ReverTra Dash kit (TOYOBO Co., Ltd., Osaka, Japan). Aliquots of total cDNA were amplified with Go Taq Green Master Mix (Promega), and amplifications for COX-2 and TNF-α were performed in a MyCycler thermal cycler (BIO-RAD, Tokyo, Japan) for 30 cycles (30 s denaturation at 94°C, 30 s annealing at 58°C, and 1 min extension at 72°C) for all primers. GAPDH was used as an internal control. The amplification reaction products were resolved on 1.5% agarose/TAE gels (Nacalaitesque, Inc., Kyoto, Japan), electrophoresed at 100 mV, and visualized by ethidium-bromide staining. PCR primer pairs were COX-2: 5′-TGAGCATCTACGGTTTGCTG-3′ (Forward) and 5′-TGCTTGTCTGGAACAACTGC-3′ (Reverse); TNF-α: 5′-TCCTTCAGACACCCTCAACC-3′ (Forward) and 5′-AGGCCCCAGTTTGAATTCTT-3′ (Reverse); GAPDH: 5′-TGAACGGGAAGCTCACTGG-3′ (Forward) and 5′-TCCACCACCCTGTTGCTGTA-3′ (Reverse).

### Western blotting

After 12 h of pre-incubation with or without palmitate (50 µM), cells were treated with *Pg*-LPS (100 ng/ml) for another 12 h. Cells were also pretreated by TLR4 signaling inhibitor Cli-095 (InvivoGen, San Diego, CA, USA) for 6 h and stimulated by palmiatate (50 µM) for indicated time. Primary antibodies against Polyclonal anti-poly (ADP-ribose) polymerase (PARP), P-p65, P-p38, P-JNK and P-ERK were purchased from Cell Signaling Technology, Inc. (Danvers, MA, USA). Monoclonal anti-β-actin was purchased from Sigma-Aldrich Co. LLC. (St. Louis, MO, USA) Western blotting was performed as previously described [18]. Cell pellets (1.5×10^5^ cells) were resuspended in ice-cold lysis buffer. Proteins were separated by SDS-PAGE and electro-blotted onto a nitrocellulose membrane. After blocking of the membrane with 3% milk for 30 min, membranes were incubated with primary antibodies (1∶1000) and anti-rabbit secondary antibody. β-actin (1∶10,000) was used as internal control. The results were visualized by Amersham ECL western blotting detection system (GE Healthcare, Japan).

### Immunofluorescent staining

HuhT1 cells grown on coverslips were fixed in 4% paraformaldehyde for 10 min at room temperature, rinsed three times with ice-cold PBS and then permeabilized in 0.1% Triton X-100 in PBS for 10 min at room temperature. After rinsing three times with PBS, the coverslips were incubated with primary antibody against *Pg* in 10% DMEM for 2 h at room temperature, rinsed three times with PBS and then incubated with anti-rabbit Alexa488 (1∶1000, Molecular Probes, Eugene, OR, USA), which functioned as the secondary antibody. DNA was visualized by DAPI staining (blue) and F-actin was visualized by Phalloidin staining (red). Pictures were recorded using Keyence BZ-8100 series All-in-one Fluorescence Microscope (KEYENCE Japan, Osaka, Japan).

### Statistical analysis

Results are reported as mean ± standard deviation (SD). Intergroup statistical differences were evaluated by student t-test or Mann-Whitney test, with the level of significance set at P<0.01 and P<0.05.

## Results

### Dental *Pg* infection induces periapical granuloma (Figure 1)

**Figure 1:**
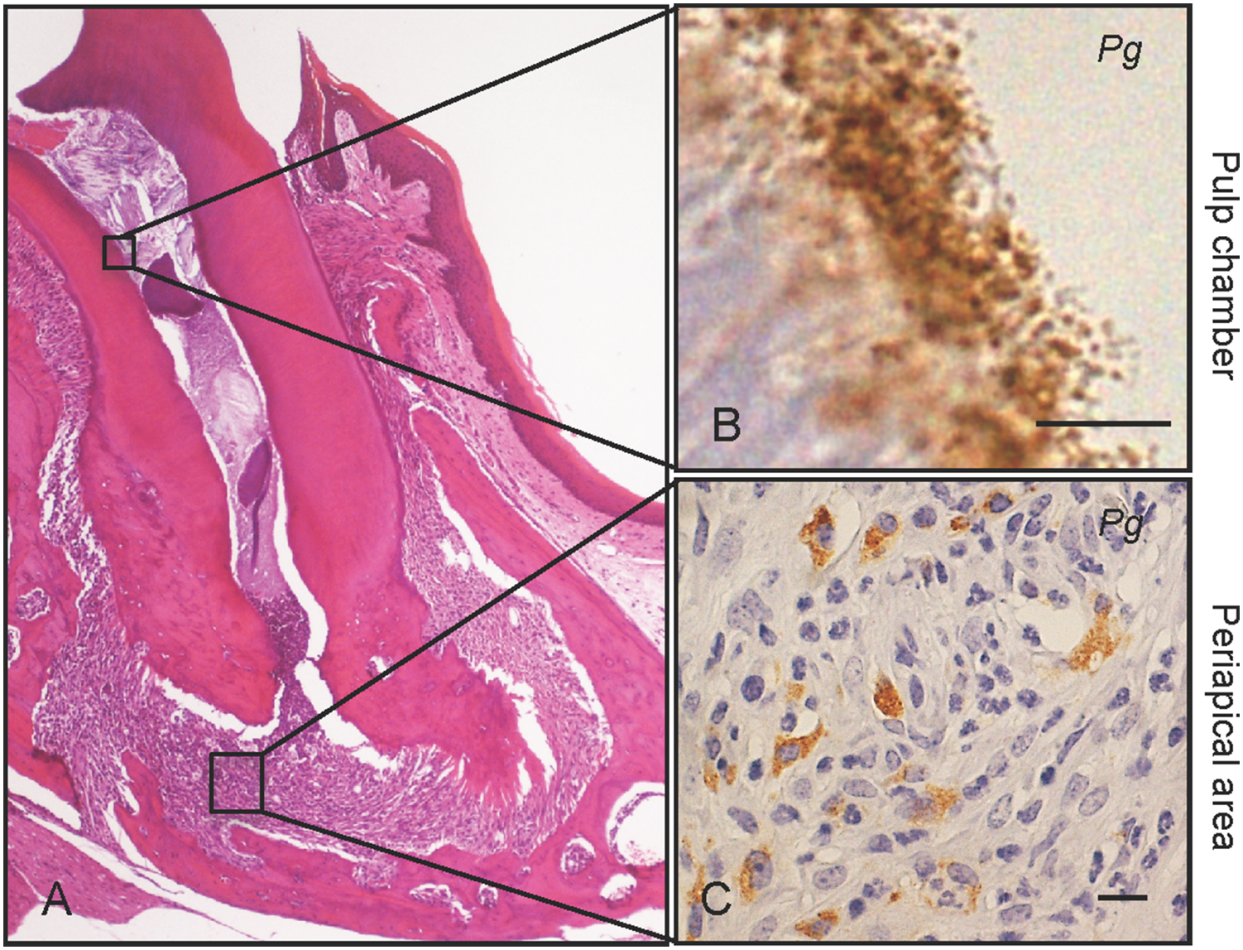
Dental *Pg* infection induces periapical granuloma. Severe pulp necrosis is observed in the first upper molars after six weeks of *Pg* infection. (A) Periapical granuloma is a representative histological feature of *Pg* infected molars, and is infiltrated with neutrophils and macrophages. (B) Immunohistochemical staining shows *Pg* colonies (brown pigment) in the pulp chambers and (C) in neutrophils and macrophages in the periapical area. Scale bar, 10 µm. *Pg, Porphyromonas gingivalis.* Experiments were performed three times or more with similar results.

In both groups of *Pg* infected mice, CD-*Pg* and HFD-*Pg*, severe pulp necrosis was observed in the first upper molars six weeks after infection. Periapical granulomas were apparent, and pulp chambers were infiltrated with neutrophils and macrophages (Figure 1A). *Pg* colonies were located in infected pulp chambers (Figure 1B), and bacteria were also present in associated neutrophils and macrophages (Figure 1C).

### *Pg* exacerbates HFD-induced endothelial injury and apoptosis (Figure 2)

**Figure 2:**
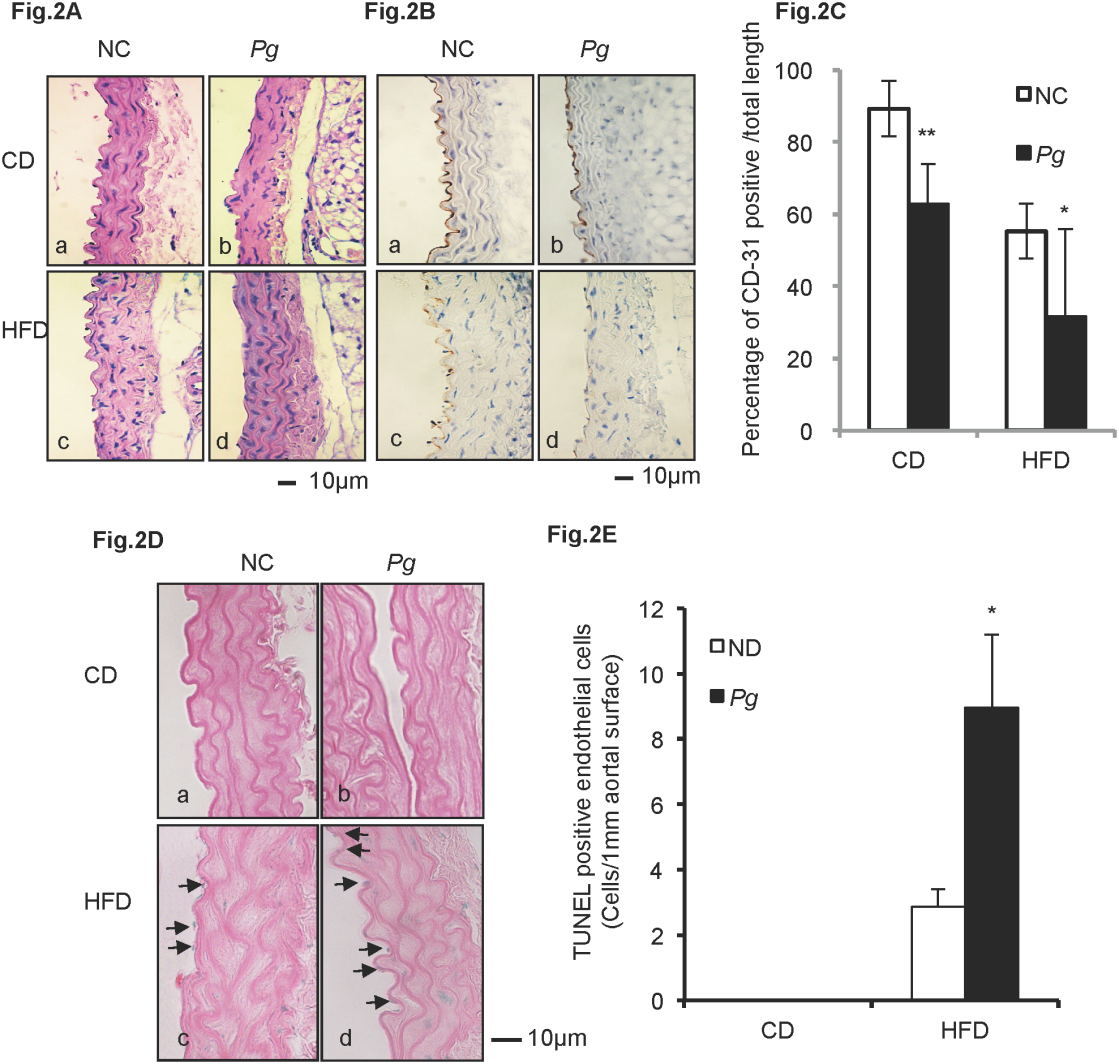
*Pg* exacerbates HFD-induced endothelial injury and apoptosis. (A) Histological findings in aortal tissue in CD mice (a, b) and in HFD mice (c, d). H&E, scale bar, 100 µm. (B) IHC staining of CD-31 (endothelial cell marker) in aortal tissue in CD mice (a, b) and in HFD mice (c, d). Scale bar, 10 µm. (C) Percentage of aortal surface covered by CD31-positive endothelial cells in the HFD-*Pg* group is significantly lower than that of the HFD-NC group. Mean ± SD *P<0.05, **P<0.01. (D) TUNEL staining of aortal tissue in CD mice (a, b) and in HFD mice (c, d). Scale bar, 10 µm. (E) Number of TUNEL-positive cells per unit length of aortal surface in HFD-*Pg* group is significantly higher than that of HFD-NC group. Mean ± SD *P<0.05. CD, chow diet; HFD, high fat diet; NC, negative control; *Pg, Porphyromonas gingivalis.* Experiments were performed three times or more with similar results.

The inner surface of aortal wall is lined by endothelial cells, and no obvious histological changes in aortal walls were apparent among the experimental groups (Figure 2Aa–d). Immunolocalization of endothelial marker CD-31 was used to further assess the condition of endothelium. In the CD-NC group, aortal walls were lined with endothelial cells exhibiting a strong positive reaction for CD-31 (Figure 2Ba), while CD-31 staining of CD-*Pg* aorta was relatively weaker (Figure 2Bb). Furthermore, CD-31 staining was further reduced and more heterogeneous in the HFD-NC group, indicating damage to the endothelial layer (Figure 2Bc). *Pg* infection further exacerbated this effect, as CD-31 expression was observed to be much weaker and discontinuous in HFD-*Pg* (Figure 2B-d). These observations were supported by quantitative analysis of the ratio of CD31-positive endothelial surface over the total length measured (Figure 2C). CD31-positive surfaces were significantly reduced in HFD groups compared to CD groups and *Pg* infection significantly reduced CD-31 positive surfaces in both the CD (P<0.01) and HFD (P<0.05) groups.

TUNEL staining was used to detect apoptosis in cells of the aortal tissues. No positive reactions were observed in cells in the CD-NC and CD*-Pg* groups (Figure 2Da, b). In HFD groups, TUNEL-positive nuclei were seen in endothelial cells and smooth muscle cells of the aortal wall. The positive reaction in HFD-*Pg* was stronger than that in HFD-NC (Figure 2Dc, d), and the number of TUNEL positive cells was significantly higher in HFD-*Pg* versus HFD-NC (P<0.05) (Figure 2E).

### Dental *Pg* infection predisposes to increased invasion of injured mouse aortal tissue under HFD conditions (Figure 3)

**Figure 3:**
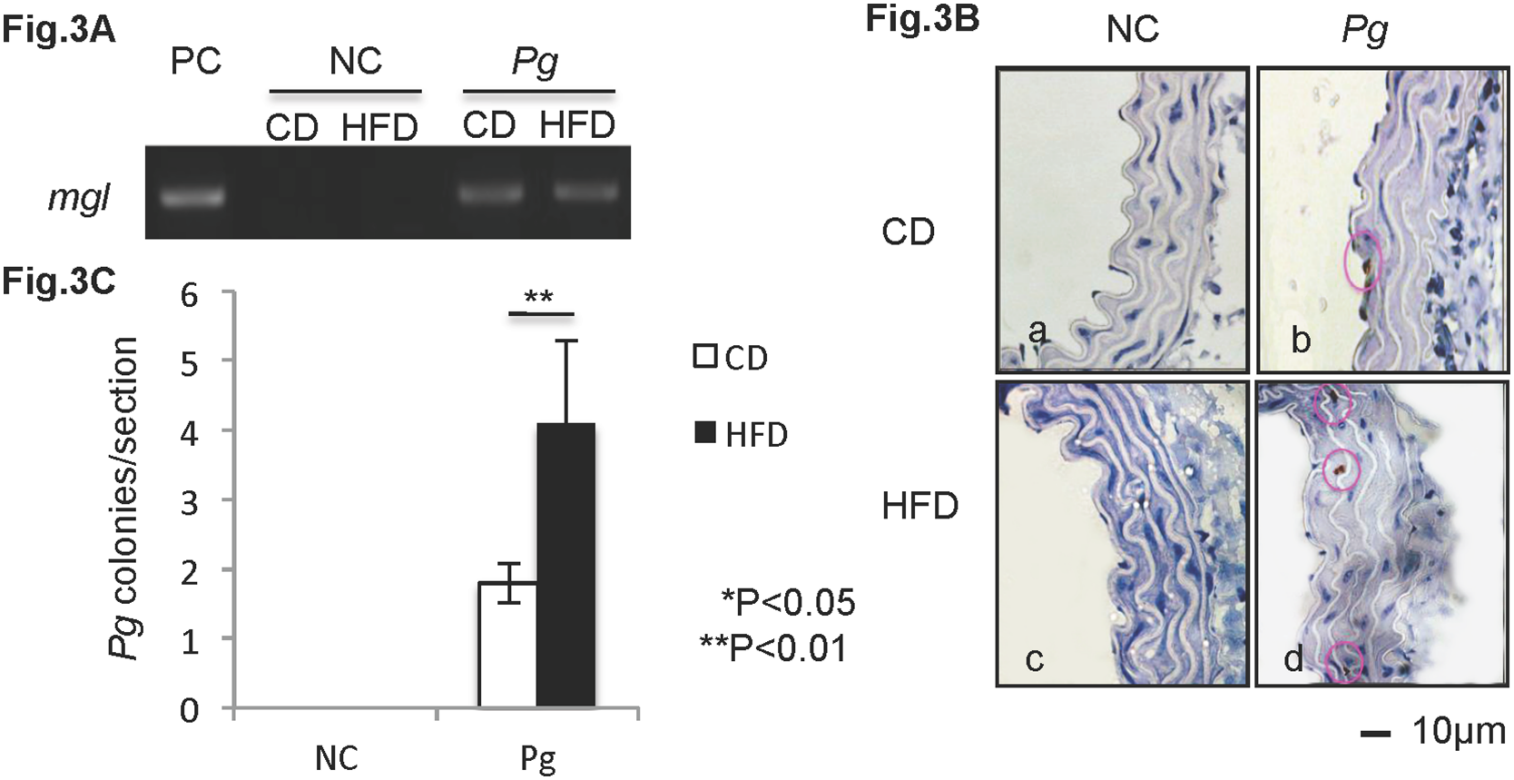
Dental *Pg* infection predisposes to increased invasion of injured mouse aortal tissue under HFD conditions. (A) Expression of the mgl gene of *Pg* is detected in aortal tissue of infected mice. Genomic DNA of *Pg* W83 strain is included as a positive control (PC). (B) Immunolocalization of *Pg* (brown particles) is observed in aortal walls of infected mice. (C) *Pg* invasion is significantly higher in HFD-*Pg* group. Mean ± SD *P<0.05, **P<0.01. Scale bar, 10 µm; CD, chow diet; HFD, high fat diet; NC, negative control; *Pg, Porphyromonas gingivalis.* Experiments were performed three times or more with similar results.

In these studies, we induced periapical periodontitis by application of live *Pg* cultures into root canals of mouse first molars. Interestingly, *Pg* DNA and *Pg* colonies were detected in aortal tissues at 6 weeks post treatment (Figure 3A and B). In Figure 3A, the *Pg*-specific *mgl* gene was detected by PCR in DNA harvested from the aortal wall of both CD-*Pg* and HFD-*Pg* groups, but not in NC groups. In the *Pg*-infected CD group, *Pg* colonies were immunohistochemically detected in the surface layer of the intima (Figure 3Bb), whereas in the HFD group, *Pg* could be observed in the deeper smooth muscle layer of the aorta (Figure 3Bd). Moreover, the number of *Pg* colonies detected in aorta sections was significantly higher (P<0.01) in the HFD group versus the CD group (Figure 3C).

### *Pg* and *Pg*-LPS increase palmitate-induced apoptosis in HuhT1 cells (Figure 4)

**Figure 4:**
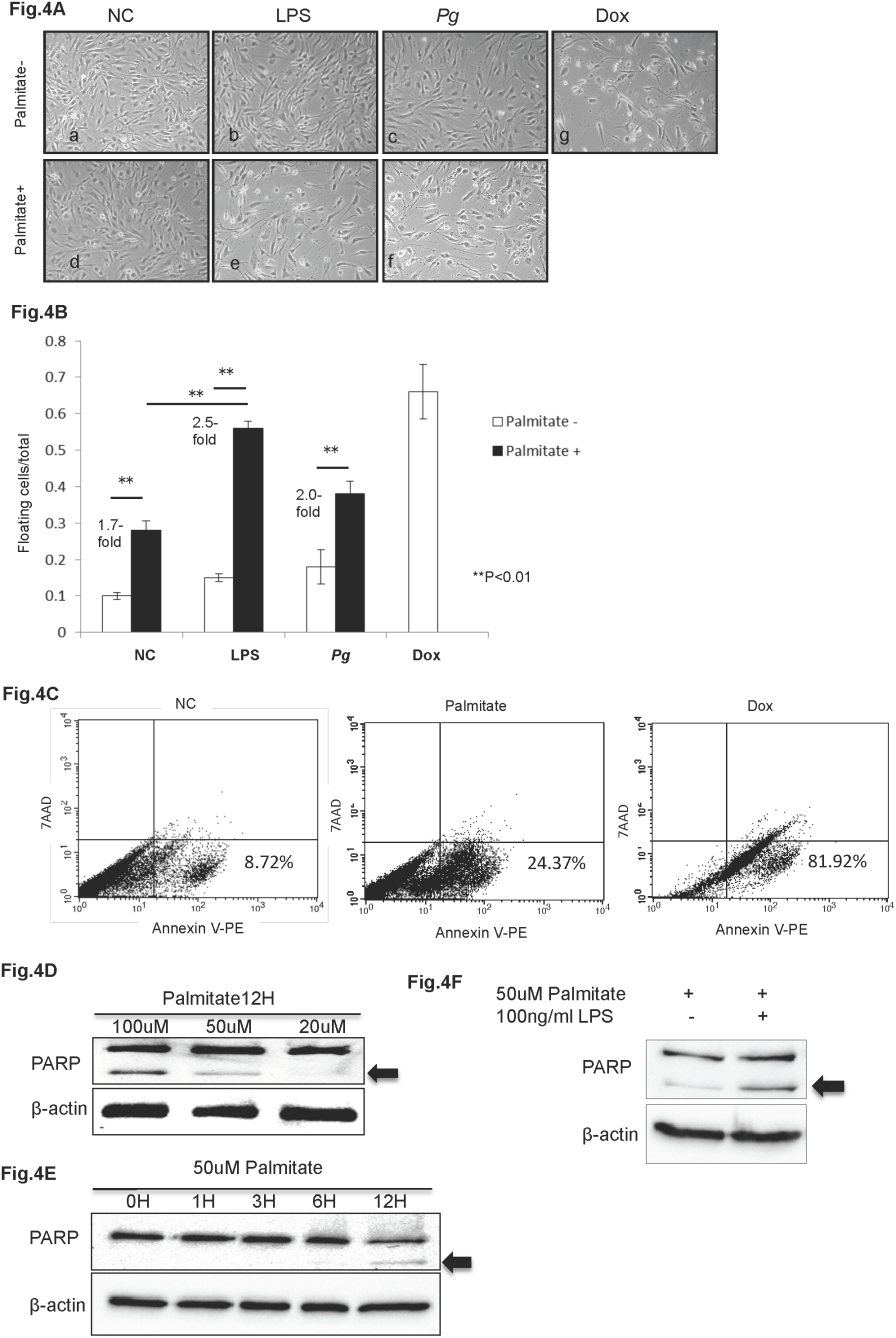
*Pg* and *Pg*-LPS increase palmitate-induced apoptosis in HuhT1 cells. (A, B) Palmitate treatment induces HuhT1 cell detachment, and *Pg* and/or *Pg*-LPS treatment further increases detachment. (C) Palmitate stimulation induces cell death by apoptosis that is indicated by increased percentage of Annexin V positive cells; Dox is positive control. (D, E) Palmitate stimulation induces PARP cleavage (arrow, cleaved PARP) in HuhT1 cells in a dose- and time-dependent manner. (F) *Pg*-LPS accelerates PARP cleavage in palmitate-treated cells. Mean ± SD, **P<0.01. NC, negative control; *Pg, Porphyromonas gingivalis*; PARP, Poly (ADP-ribose) polymerase; Dox, Doxorubicin. Experiments were performed three times or more with similar results.

We used hTERT-immortalized human umbilical vein endothelial cells (HuhT1) as a model to explore the effects of *Pg*-LPS *in vitro*. Previously, we demonstrated up-regulation of serum LPS in both CD-*Pg* and HFD-*Pg* groups [20]. Therefore, we examined the combined effects on HuhT1cells of *Pg*-LPS and palmitate, a representative FFA that is elevated as a result of obesity-induced endothelial injury. In cells untreated by palmitate, there were no significant morphological changes in HuhT1 cells, except for slight cell shrinkage after *Pg*-LPS or *Pg* whole cell treatment (Figure 4A a, b, c). In contrast, palmitate treatment caused marked cell shrinkage and triggered cells to detach from dishes (Figure 4Ad). Palmitate pre-treatment followed by addition of *Pg*-LPS or *Pg* whole cell induced more severe cell detachment, however, the effect of Pg whole cell was not as robust as Pg-LPS (Figure 4A e, f). Quantification of these observations confirmed that in the NC group, palmitate treatment induced a 1.7-fold increase in floating cells compared to the untreated group (P<0.01). *Pg*-LPS stimulation alone did not induce significant cell detachment, whereas, *Pg*-LPS in combination with palmitate induced a significant 2.5-fold increase in floating cells over palmitate-untreated cells (P<0.01). Similarly, *Pg* whole cell treatment induced 2.0-fold increase in floating cells with palmitate+ vs palmitate- treatment (Figure 4B). Flow cytometry detected 24.37% of cells treated by palmitate undergoing early apoptosis with higher affinity to Annexin V compared to 8.72% in non-treated cells (Figure 4C). Dox served as positive control (Figure 4A, 4B, 4C).

Poly (ADP-ribose) polymerase (PARP) cleavage is an established and reliable apoptosis indicator downstream of caspase family activation, especially caspase 3 and caspase 7. To determine potential induction of apoptosis of HuhT1 cells by palmitate treatment, we examined PARP cleavage at the protein level. After 12 h of palmitate stimulation, a dose-dependent PARP cleavage was observed, detectable at the low dose of 50 µM (Figure 4D). A time course revealed that PARP was cleaved by 12 h after 50 µM palmitate treatment (Figure 4E), a time point at which substantial numbers of cells were observed to be detached (data not shown) and likely undergoing apoptosis. Furthermore, *Pg*-LPS treatment increased palmitate-induced PARP cleavage (Figure 4F).

### *Pg* and *Pg*-LPS up-regulate COX-2 and TNF-α expression in palmitate treated HuhT1 cells via activated TLR4 (Figure 5)

**Figure 5:**
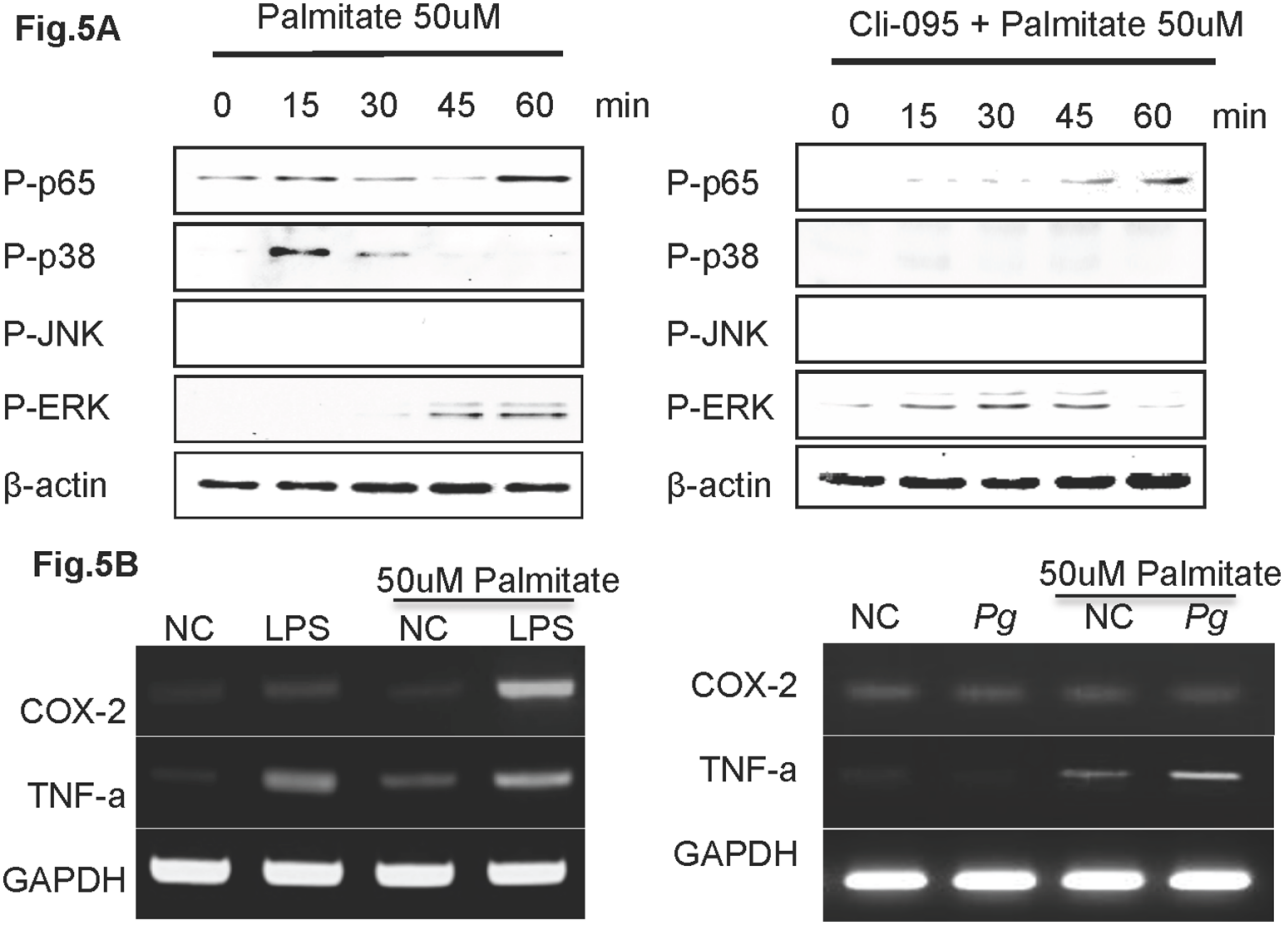
*Pg*-LPS up-regulates COX-2 and TNF-α expression in palmitate treated HuhT1 cells. (A) Phosphorylation of p65, p38, JNK and ERK was detected by western blotting. Cells were pretreated with Cli-095 6 h before palmitate stimulation. (B) mRNA expression of COX-2 and TNF-α in HuhT1 cells with and without palmitate treatment at 1 h after Pg and/or *Pg*-LPS stimulation. NC, negative control; *Pg, Porphyromonas gingivalis*; Cli-095, TLR4 inhibitor. Experiments were performed three times or more with similar results.

We next investigated effects of palmitate priming on cells. Palmitate induced phosphorylation of p65 and p38 as early as 15 min and ERK at 45 min after treatment. 6 h pretreatment Toll-like receptor-4 (TLR4) inhibitor, Cli-095, prevented phosphorylation of p65 and p38 at early time point induced by palmitate (Figure 5A). Effects of *Pg*-LPS on expression of inflammation associated factors COX-2 (a synthetic enzyme of inducible PGE_2_) and TNF-α. *Pg*-LPS induced a slight increase in COX-2 mRNA, and a more robust increase in TNF-α in HuhT1 cells Palmitate treatment alone increased TNF-α expression. Unexpectedly, 1 h of *Pg* whole cell priming on HuhT1 cells did not alter the mRNA expression of COX-2 and TNF-α. In the palmitate pre-treated group, expression of both COX-2 and TNF-α were strongly enhanced by *Pg*-LPS stimulation and only TNF-α mRNA was increased by Pg whole cell stimulation (Figure 5B).

### Palmitate increases *Pg* cell invasion in HuhT1 cells (Figure 6)

**Figure 6:**
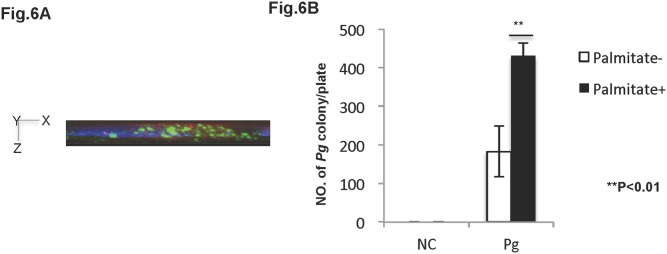
Palmitate increases *Pg* cell invasion in HuhT1 cells. Antibody protection assay (see Material & Methods) was used to determine *Pg* invasion in HuhT1 cells. (A) Invaded *Pg* was analyzed by fluorescent microscopy at magnification of 1000x. Side view (X–Z plane) is shown. *Pg* (green); DAPI (blue); Phalloidin (red). (B) *Pg* invasion into HuhT1 cells is increased under palmitate pre-treatment. Mean ± SD, **P<0.01. NC, negative control; *Pg Porphyromonas gingivalis*. Experiments were performed three times or more with similar results.

The antibiotic protection assay was performed to determine whether *Pg* cells were capable of invading and remaining viable within HuhT1 cells, *in vitro*. Immunofluorescent staining exhibited the existence of invaded *Pg* shown in green in HuhT1 cells (Figure 6A). Brown *Pg* colonies appeared 5 days post plating on blood agar (data not shown). Pretreatment of cells with 50 µM palmitate significantly increased the number of invading *Pg* colonies in HuhT1 cells (P<0.01) (Figure 6B).

## Discussion

The health of the inner arterial wall is an important factor in preventing vascular disease development. However, the endothelium is easily damaged by various atherogenic factors, including hypercholesterolemia, homocysteine, oxidative stress, and hyperglycemia [19]. To study the effect of dental infection on endothelial injury associated with obesity, we induced obesity in mice by high fat diet feeding, and then applied *Pg* bacteria to pulp chambers of upper molars.

The relationship between periodontitis and systemic disease has been the subject of intense speculation and research in recent years. Periodontitis, a bacterially induced inflammatory disease, destroys the periodontal tissues and may ultimately lead to tooth exfoliation. Among the over 700 bacterial species colonizing the oral cavity [20], *Pg* is highly detectable in the periodontal pocket during chronic periodontitis, and is considered to be one of the principal bacterial species responsible for inducing periapical periodontitis [11]–[13]. *Pg* is a Gram-negative species that requires anaerobic conditions for growth. In our mouse model, we delivered *Pg* into pulp chambers, providing a natural route for infection to induce periapical periodontitis. Dental infection by *Pg* caused total pulp necrosis and establishment of periapical granuloma, in which *Pg* was detected in neutrophils and macrophages. Previously, we have shown using this mouse model that *Pg* colonies remained viable inside infected pulp chambers and that bacteria were able to reproduce over a long period of time to induce periapical periodontitis, affecting the pathological progression of nonalcoholic steatohepatitis [21]. In this model, we hypothesize that the periapical granulomas are a stable and persistent source of the *Pg* and its immunogenic products, allowing spreading through the bloodstream.

In the mouse model used in the present study, the reduced expression of endothelial marker CD31 and increased presence of TUNEL staining revealed the loss of endothelial integrity, identifying endothelial injury in the HFD-NC group compared with the CD-NC group (Figure 2). In addition, the body weight, epididymal fat weight, and total cholesterol were elevated in HFD-fed mice (Table S1), confirming the establishment of dyslipidemia caused by obesity. Correspondingly, using the diet-induced atherosclerotic rabbit model, Yu *et al*. reported that long term (16 weeks) feeding of HFD induced loss of integrity in the endothelial layer [22]. According to Boden *et al*. FFAs appear to be a major link between obesity and the development of atherosclerotic vascular disease [23]. *In vitro* studies showed that FFA treatment would induce apoptosis of various cells, including endothelial cells [24], and accelerated apoptosis likely contributes to the loss of endothelial integrity, in turn leading to increased permeability. In these studies, we additionally demonstrate palmitate-induced higher affinity to Annexin V and PARP cleavage in HuhT1 cells (Figure 4C, 4D, 4E), indicating that increased apoptosis is a potential mechanism for FFA-induced endothelial cell layer injury.

In the HFD groups, addition of *Pg* further reduced expression of CD31 and increased presence of TUNEL positive cells in the endothelial layer, compared to the non-*Pg*-treated controls. CD31 is an adhesion molecule expressed on endothelial cells that is important for maintaining endothelial integrity [25] and facilitates transendothelial migration of neutrophils to sites of inflammation [26]. Carrithers *et al.* revealed that CD31-deficient mice showed enhanced vascular permeability and increased apoptotic endothelial cell death during LPS-induced shock, compared to wild type controls, suggesting that CD31 is necessary for maintenance of endothelial integrity and prevention of apoptosis [27]. *Pg* infection increases serum levels of LPS in our animal model [21]. In the *in vitro* portion of these studies, we observed that addition of *Pg*-LPS to HuhT1 cells significantly increased cell detachment with FFA pre-stimulation (Figure 4B). Furthermore, PARP cleavage clearly showed that *Pg*-LPS promoted cell apoptosis with FFA pre-treatment (Figure 4F). For future studies, it will be of interest to investigate the role of CD-31 in LPS- and FFA-induced endothelial cell apoptosis. Interestingly, although the HFD-*Pg in vivo* exhibited most severe endothelial injury and highest number of *Pg* colonies in aorta, the effect of *Pg* whole cell was not as robust as *Pg*-LPS in inducing endothelial cell death with addition of FFA. Yun *et al*. reported that gingipains (major virulence determinants) of *Pg* may contribute to the initiation of endothelial barrier destruction by cleaving CD31 at the endothelial cell-cell junction, eventually resulting in increased vascular permeability [28]. The *Pg* invasion in aortal wall observed in the current study may be the consequence of increased vascular permeability induced by degradation of CD31, probably through gingipains. However, a better understanding of this mechanism requires further investigation.

TNF-α is one of the key cytokines in inflammatory pathologies, stimulating the production of other proinflammatory cytokines, chemokines, and also COX-2, consequently contributing to endothelial cells apoptosis [29]. In the present study, a single *Pg*-LPS or FFA treatment increased COX-2 and TNF-α gene expression in endothelial cells. It is reported that palmitate activates NF-κB and increases inflammatory cytokine production via TLR4 in endothelial cells and adipocytes, which is consistent with our result [30], [31]. Interestingly, *Pg*-LPS stimulation dramatically up-regulated COX-2 and TNF-α gene expression in FFA-pretreated endothelial cells (Figure 5). To our knowledge, this is the first demonstration of a combined effect of *Pg*-LPS and FFA on inflammatory mediator production and endothelial cell apoptosis, though more work will be done to show whether the effect is additive or synergystic. As both FFA and LPS are ligands to TLR4 and could induce proinflammatory cytokines through the NF-кB pathway [32], [33], the cooperative effect of FFA and *Pg*-LPS on TNF-α up-regulation may be due to the co-activation of NF-кB pathway via TLR4. It is known that *in vivo*, proinflammatory cytokines produced in response to FFA contribute to low-grade inflammation in fat, liver, and the aortal wall [34], [35], and we propose that *Pg*-LPS exacerbates the inflammation in the aortal wall by further up-regulating cytokines. This is supported by the immunohistochemical staining and RT-PCR detection of *Pg* colonies in aortal wall of infected mice (Figure 3). Furthermore, we demonstrated that there is a significant difference between CD-*Pg* and HFD-*Pg* referring the tissue invasion rate by applying equal amount of *Pg* from pulp chamber (Figure 3C). Correspondingly, our *in vitro* study showed that *Pg* easily invaded damaged HuhT1 endothelial cells following FFA pretreatment. Several reports demonstrated the detection of DNA of *Pg* and *Aggregatibacter actinomycetemcomitans* (*Aa*) in atheromatous plaque, with *Pg* (78.57%) as the most commonly found periodontal pathogens followed by *Aa* (66.67%) [36]. These findings point to the capability for periodontal pathogens to access systemic circulation, colonize distant sites such as the aortal wall, and influence the pathophysiology of atherogenesis.

Based on these previous findings and data generated in the current study, we hypothesize that *Pg*-LPS may exacerbates FFA-induced endothelial injury in aortal walls of obese individuals, providing easy access for circulating *Pg* to invade and colonize the deep intima and promote the development of atherosclerosis through additive local production of pro-inflammatory cytokines. However, further investigations will help clarify the effect of *Pg* infection on endothelial cells and smooth muscle cells in the aortal wall.

In summary, data from this study demonstrate that *Pg* from a dental infection are capable of invading aortal tissue through the circulation and exacerbating HFD-induced endothelial damage, in part by accelerating endothelial cell apoptosis. On the other hand, HFD-induced endothelial damage enhanced *Pg* invasion rate. Furthermore, FFAs contributed to the up-regulation of inflammation and apoptosis of endothelial cells, and *Pg*-LPS aggravated this effect. Taken together, this is the first demonstration that dental infection of *Pg* can contribute to endothelial injury in obese mice. Extrapolation these results suggests that careful attention to dental hygiene or periodontal treatment and maintenance may be beneficial for obese patients in order to prevent or minimize adverse effects of bacterial infection on the early stages of atherosclerosis.

## Supporting Information

Table S1 **Establishment of dyslipidemia in HFD-fed mice.** Body weight, epididymal fat weight and total cholesterol were measured in four groups of mice (CD-NC, CD-*Pg*, HFD-NC and HFD-*Pg*); ^a^p<0.01 to CD-NC, ^b^p<0.01 to CD-*Pg*,^ c^p<0.05 to CD-NC and ^d^p<0.05 to CD-*Pg*. CD, chow diet; HFD, high fat diet; NC, negative control; *Pg, Porphyromonas gingivalis.* Experiments were performed three times with similar results.
(TIF)

## References

[1] Ross R, Harker L (1976) Hyperlipidemia and atherosclerosis. Science 193(4258): 1094–100. 822515 10.1126/science.822515

[2] Fuster V, Kelly BB, editors (2010) Promoting Cardiovascular Health in the Developing World: A Critical Challenge to Achieve Global Health. Washington (DC): National Academies Press (US). 20945571

[3] Libby P (2012) Inflammation in atherosclerosis. Arterioscler Thromb Vasc Biol 32(9): 2045–51 10.1161/ATVBAHA.108.179705 22895665 PMC3422754

[4] Ross R (1999) Atherosclerosis is an inflammatory disease. Am Heart J 138(5 Pt 2): S419–20. 10.1016/s0002-8703(99)70266-8 10539839

[5] Tritto I, Ambrosio G (2004) The multi-faceted behavior of nitric oxide in vascular “inflammation”: catchy terminology or true phenomenon? Cardiovasc Res 63(1): 1–4. 15194454 10.1016/j.cardiores.2004.04.028

[6] Shah PK (1999) Plaque disruption and thrombosis Potential role of inflammation and infection. Cardiol Clin 17(2): 271–81. 10384826 10.1016/s0733-8651(05)70074-6

[7] Libby P, Egan D, Skarlatos S (1997) Roles of infectious agents in atherosclerosis and restenosis: an assessment of the evidence and need for future research. Circulation 96(11): 4095–103. 9403635 10.1161/01.cir.96.11.4095

[8] Kuvin JT, Kimmelstiel CD (1999) Infectious causes of atherosclerosis. Am Heart J 137(2): 216–26. 9924154 10.1053/hj.1999.v137.92261

[9] Wang Z, Zhang M, Yu Z, Shui Y, Ding Y, et al (2012) An animal experiment study on the effect of periodontitis on atherosclerosis. Hua Xi Kou Qiang Yi Xue Za Zhi 30(3): 308–13. 22768774

[10] Cai Y, Kurita-Ochiai T, Hashizume T, Yamamoto M (2012) Green tea epigallocatechin-3-gallate attenuates Porphyromonas gingivalis-induced atherosclerosis. FEMS Immunol Med Microbiol Sep 11. doi:10.1111/1574-695x.12001. 10.1111/2049-632X.12001 23620122

[11] Yang HW, Huang YF, Chou MY (2004) Occurrence of Porphyromonas gingivalis and Tannerella forsythensis in periodontally diseased and healthy subjects. J Periodontol 75: 1077–1083. 15455734 10.1902/jop.2004.75.8.1077

[12] Saito D, Coutinho LL, Borges Saito CP, Tsai SM, Hofling JF (2009) Real-time polymerase chain reaction quantification of Porphyromonas gingivalis and Tannerella forsythia in primary endodontic infections. J Endod 35(11): 1518–24 10.1016/j.joen.2009.08.005 19840640

[13] Pereira CV, Stipp RN, Fonseca DC, Pereira LJ, Hofling JF (2001) Detection and clonal analysis of anaerobic bacteria associated to endodontic-periodontal lesions. J Periodontol 82: 1767–1775 10.1902/jop.2011.110063. Epub 2011 Mar 29 21513472

[14] Yoshimura M, Nakano Y, Yamashita Y, Oho T, Saito T (2000) Formation of methyl mercaptan from L-methionine by Porphyromonas gingivalis. Infect Immun 68: 6912–6916. 11083813 10.1128/iai.68.12.6912-6916.2000 PMC97798

[15] Anno K, Hayashi A, Takahashi T, Mitsui Y, Ide T, et al.. (2007) Telomerase activation induces elongation of the telomeric single-stranded overhang, but does not prevent chromosome aberrations in human vascular endothelial cells. Biochem Biophys Res Commun. 23; 353(4): 926–32. Epub 2006 Dec 22. 10.1016/j.bbrc.2006.12.112 17207773

[16] Wobser H, Dorn C, Weiss TS, Amann T, Bollheimer C, et al (2009) Lipid accumulation in hepatocytes induces fibrogenic activation of hepatic stellate cells. Cell Res 19(8): 996–1005 10.1038/cr.2009.73 19546889

[17] Lamont RJ, Chan A, Belton CM, Izutsu KT, Vasel D, et al (1995) Porphyromonas gingivalis invasion of gingival epithelial cells. Infect Immun 63: 3878–3885. 7558295 10.1128/iai.63.10.3878-3885.1995 PMC173546

[18] Inubushi T, Kawazoe A, Miyauchi M, Kudo Y, Ao M, et al.. (2012) Molecular mechanisms of the inhibitory effects of bovine lactoferrin on lipopolysaccharide-mediated osteoclastogenesis. J Biol Chem 287(28): 2 3527–36. doi:10.1074/jbc.M111.324673. Epub 2012 May 16. 10.1074/jbc.M111.324673 PMC3390628 22593578

[19] Fernandez AZ, Siebel AL, El-Osta A (2010) Atherogenic factors and their epigenetic relationships. Int J Vasc Med 2010: 437809 10.1155/2010/437809. Epub 2010 Sep 16 21152193 PMC2989709

[20] Aas JA, Paster BJ, Stokes LN, Olsen I, Dewhirst FE (2005) Defining the normal bacterial flora of the oral cavity. J Clin Microbiol 43(11): 5721–32. 16272510 10.1128/JCM.43.11.5721-5732.2005 PMC1287824

[21] Furusho H, Miyauchi M, Hyogo H, Inubushi T, Ao M, et al.. (2013) Dental infection of Porphyromonas gingivalis exacerbates high fat diet-induced steatohepatitis in mice. J Gastroenterol Jan 11. DOI 10.1007/s00535-012-0738-1. 10.1007/s00535-012-0738-1 23307045

[22] Yu Q, Li Y, Waqar AB, Wang Y, Huang B, et al (2012) Temporal and quantitative analysis of atherosclerotic lesions in diet-induced hypercholesterolemic rabbits. J Biomed Biotechnol 2012: 506159 10.1155/2012/506159. Epub 2012 Mar 14 22505812 PMC3312324

[23] Boden G (2008) Obesity and free fatty acids. Endocrinol Metab Clin North Am 37(3): 635–46, viii–ix. doi:10.1016/j.ecl.2008.06.007. 10.1016/j.ecl.2008.06.007 PMC2596919 18775356

[24] Piro S, Spampinato D, Spadaro L, Oliveri CE, Purrello, et al. (2008) Direct apoptotic effects of free fatty acids on human endothelial cells. Nutr Metab Cardiovasc Dis 18(2): 96–104. Epub 2007 Jun 8. 10.1016/j.numecd.2007.01.009 17560770

[25] Graesser D, Solowiej A, Bruckner M, Osterweil E, Juedes A, et al (2002) Altered vascular permeability and early onset of experimental autoimmune encephalomyelitis in PECAM-1-deficient mice. J Clin Invest 109: 383–392. 11827998 10.1172/JCI13595 PMC150854

[26] Muller W, Weigl S, Deng X, Phillips D (1993) PECAM-1 is required for transendothelial migration of leukocytes. J Exp Med 178: 449–460. 8340753 10.1084/jem.178.2.449 PMC2191108

[27] Carrithers M, Tandon S, Canosa S, Michaud M, Graesser D, et al (2005) Enhanced susceptibility to endotoxic shock and impaired STAT3 signaling in CD31-deficient mice. Am J Pathol Jan 166(1): 185–96. 10.1016/S0002-9440(10)62243-2 PMC1602311 15632011

[28] Yun PL, Decarlo AA, Chapple CC, Hunter N (2005) Functional implication of the hydrolysis of platelet endothelial cell adhesion molecule 1 (CD31) by gingipains of Porphyromonas gingivalis for the pathology of periodontal disease. Infect Immun. Mar 73(3): 1386–98. 10.1128/IAI.73.3.1386-1398.2005 PMC1064963 15731036

[29] Filippatos G, Ang E, Gidea C, Dincer E, Wang R, et al (2004) Fas induces apoptosis in human coronary artery endothelial cells in vitro. BMC Cell Biol 22 5: 6. 10.1186/1471-2121-5-6 PMC331398 14738570

[30] Maloney E, Sweet IR, Hockenbery DM, Pham M, Rizzo NO, et al (2009) Activation of NF-kappaB by palmitate in endothelial cells: a key role for NADPH oxidase-derived superoxide in response to TLR4 activation. Arterioscler Thromb Vasc Biol. 2009 Sep 29(9): 1370–5. 10.1161/ATVBAHA.109.188813 PMC2775080 19542021

[31] Ajuwon KM1, Spurlock ME (2005) Palmitate activates the NF-kappaB transcription factor and induces IL-6 and TNFalpha expression in 3T3-L1 adipocytes. J Nutr. 2005 Aug 135(8): 1841–6. 10.1093/jn/135.8.1841 16046706

[32] Collins T, Read M, Neish A, Whitley M, Thanos D, et al (1995) Transcriptional regulation of endothelial cell adhesion molecules: NF- kappa B and cytokine-inducible enhancers. FASEB J 9: 899–909. 7542214

[33] Liu SF, Malik AB (2006) NF-kappa B activation as a pathological mechanism of septic shock and inflammation. Am J Physiol Lung Cell Mol Physiol 290(4): L622–L645. 16531564 10.1152/ajplung.00477.2005

[34] Kershaw EE, Flier JS (2004) Adipose tissue as an endocrine organ. J Clin Endocrinol Metab 89(6): 2548–56. 15181022 10.1210/jc.2004-0395

[35] Xu H, Barnes GT, Yang Q, Tan G, Yang D, et al (2003) Chronic inflammation in fat plays a crucial role in the development of obesity-related insulin resistance. J Clin Invest 112(12): 1821–30. 14679177 10.1172/JCI19451 PMC296998

[36] Figuero E, Sánchez-Beltrán M, Cuesta-Frechoso S, Tejerina JM, del Castro JA, et al (2011) Detection of periodontal bacteria in atheromatous plaque by nested polymerase chain reaction. J Periodontol 82(10): 1469–77 10.1902/jop.2011.100719. Epub 2011 Mar 29 21453047

