# Supplementary figures and images for: Infection with Porphyromonas gingivalis Exacerbates Endothelial Injury in Obese Mice
Source: PLoS One. 2014 Oct 21;9(10):e110519. doi: 10.1371/journal.pone.0110519 (PMC4204882; doi:10.1371/journal.pone.0110519)

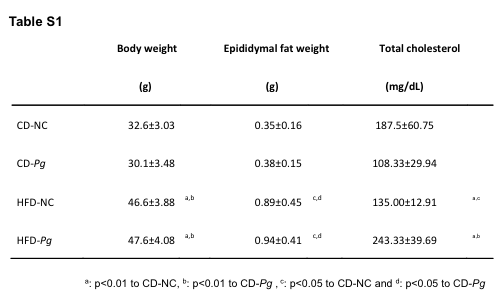

Supplement: Table S1 — Establishment of dyslipidemia in HFD-fed mice. Body weight, epididymal fat weight and total cholesterol were measured in four groups of mice (CD-NC, CD-Pg, HFD-NC and HFD-Pg); ap<0.01 to CD-NC, bp<0.01 to CD-Pg, cp<0.05 to CD-NC and dp<0.05 to CD-Pg. CD, chow diet; HFD, high fat diet; NC, negative control; Pg, Porphyromonas gingivalis. Experiments were performed three times with similar results. (TIF) [file pone.0110519.s001.tif]
